# Subjective perception of activity level: A prognostic factor for developing chronic dizziness after vestibular schwannoma resection?

**DOI:** 10.3389/fneur.2022.925801

**Published:** 2022-08-19

**Authors:** Lien Van Laer, Ann Hallemans, Vincent Van Rompaey, Claudia De Valck, Paul Van de Heyning, Luc Vereeck

**Affiliations:** ^1^Department of Rehabilitation Sciences and Physiotherapy/Movant, Faculty of Medicine and Health Science, University of Antwerp, Antwerp, Belgium; ^2^Multidisciplinary Motor Centre Antwerp (M^2^OCEAN), University of Antwerp, Antwerp, Belgium; ^3^Department of Otorhinolaryngology and Head and Neck Surgery, Faculty University Hospital of Antwerp of Medicine and Health Sciences, University of Antwerp, Antwerp, Belgium; ^4^Department of Otorhinolaryngology and Head and Neck Surgery, General Hospital Turnhout, Turnhout, Belgium

**Keywords:** vestibular schwannoma, physical activity, chronic dizziness, balance, risk factors

## Abstract

**Introduction:**

A vestibular schwannoma (VS) resection causes an acute unilateral vestibular deafferentation resulting in acute postoperative symptoms. Despite the expected resolution of most of the symptoms, due to central vestibular compensation, more than one out of four patients develop chronic dizziness. Several predictive factors, such as age and tumor size, have been suggested. Despite its potential effect on the process of central vestibular compensation, the level of physical activity after VS resection was not yet considered. Therefore, the association between the level of physical activity and chronic dizziness after VS resection will be investigated.

**Methods:**

This retrospective cohort study included 66 patients who underwent a retro-sigmoid VS resection between October 2001 and February 2007. Patients were assessed before surgery and at 9 weeks and 6 months postoperatively. At 9 weeks, patients were asked to report their level of physical activity (PA) during the past week by using a visual analogue scale and their balance performance was assessed by four standing balance conditions with eyes closed and the Timed Up and Go test (TUG). Based on the Dizziness Handicap Inventory (DHI) score at 6 months, patients were divided in a chronic dizziness group (DHI > 30) and non-chronic dizziness group (DHI-score ≤ 30). Age, sex, Koos classification, preoperative vestibular function, treatment group, balance performance, and level of PA were compared between both groups and used as independent variables in linear regression analyses with the DHI score at 6 months as dependent variable.

**Results:**

The chronic dizzy patients revealed to have significantly lower levels of PA (*p* < 0.001) and worse static and dynamic balance performance (*p* = 0.023 and *p* = 0.041, respectively) 9 weeks after surgery. After elimination, the multiple regression analysis resulted in a model with two variables (PA level, TUG) which significantly predicted the DHI score (*F*_2,42_ = 6.581; *R*^2^ = 0.239; *p* = 0.003).

**Conclusion:**

This study revealed associations between (1) the level of PA and balance performance in the subacute phase and (2) chronic dizziness after VS resection. Assessment of the level of PA and balance performance during the subacute phase, which can be performed in a non-invasive and non-time-consuming way, might therefore provide prognostic information after VS resection.

## Introduction

Vestibular schwannomas (VS) are one of the most common intracranial benign tumors, representing over 80% of cerebellopontine angle tumors (1–3). Symptoms such as hearing loss, tinnitus, vertigo, and/or neuropathies can be present, depending on tumor size and location (3). Different treatments can be considered, of which observation with annual follow-up, resection surgery and radiosurgery or therapy are the most prevailing options (3–5). A VS resection causes an acute unilateral vestibular deafferentation so that postoperative symptoms such as acute dizziness, unsteadiness, and nausea may occur to a greater or lesser extent, depending on the residual vestibular function before surgery (6–10). In case of sudden vestibular function loss, central vestibular compensation is expected to take place after the acute phase, leading to a balanced sensory reweighting and resolution of the majority of the symptoms (11, 12). Despite the expected process of central vestibular compensation, more than one out of four patients develop persistent symptoms of dizziness (28%) (7). When investigating possible influencing factors for developing chronic dizziness, conflicting evidence was found regarding associations between chronic dizziness and age, tumor size, and preoperative vestibular function (7, 13, 14). These factors thus only partially explain the variation in outcome concerning chronic dizziness after VS resection. Another potential influencing factor is the level of physical activity, as it is assumed that repetition of movement is needed to stimulate central vestibular compensation (15–19). The preoperative level of physical activity was previously investigated and it was concluded that a higher level of physical activity before surgery led to better balance performance after VS resection (20, 21). Balance performance was used in these studies as an outcome measure. However, poorer balance performance during the subacute phase might indicate development into chronic dizziness, as an association between balance performance and dizziness in vestibulopathies has previously been reported (22–24). Despite this relation, balance performance was not yet considered as an influencing factor for chronic dizziness after a VS resection. The same applies to the postoperative level of physical activity, which can be more challenging for the patient compared to before surgery, as initially (head) movements will provoke symptoms due to the acute deafferentation (16). This was confirmed by two studies that found a lower variability in head movements, and thus an altered head movement strategy, during gait tasks at 6 weeks after VS resection compared to before (25, 26). Furthermore, patients with chronic vestibulopathies have lower physical activity levels compared to healthy controls (27–29). These results suggest that although physical activity is recommended to stimulate central vestibular compensation, patients might avoid (head) movements. Revealing an association between the level of physical activity and chronic dizziness could lead to additional clinically relevant postoperative measurements and ultimately to changes in the management of patients after a VS resection. Therefore, the hypotheses of this study are whether (1) the subjective level of physical activity differs between chronic dizzy and non-chronic dizzy patients and (2) the subjective level of physical activity influences the development of chronic dizziness.

## Materials and methods

### Participants

A retrospective cohort study was performed on patients who underwent VS resection *via* a retrosigmoid approach (30) at the Antwerp University Hospital. In the period between 19 October 2001 and 23 February 2007, patient records for which a Dizziness Handicap Inventory (DHI) measurement was available 6 months after surgery were included in the study (31). This study was approved by the ethical committee of the Antwerp University Hospital and the University of Antwerp (18/13/182).

### Outcome measures

**Figure 1:**
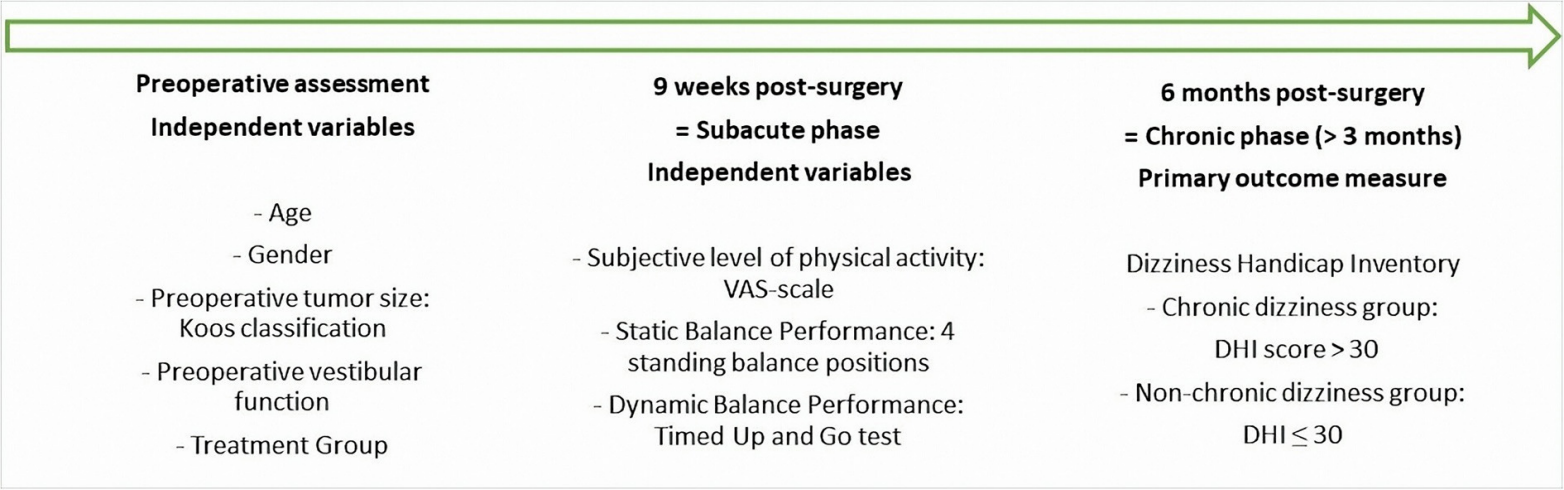
Timeline clinical assessments.

The objective of this study was to identify influencing factors for developing chronic dizziness after a VS resection. Therefore, the relationship between perceived handicap due to dizziness at 6 months after surgery (dependent variable) and the following independent variables was studied: age, sex, tumor size, preoperative vestibular function, level of physical activity, and balance performance during the subacute phase. At the time, for other research purposes, all patients were given a specific type of treatment. Therefore, treatment protocol was considered an independent variable as well. A complete overview of the clinical assessments performed at different timepoints is presented in Figure 1.

#### Perceived handicap due to dizziness/instability

The primary outcome measure of this study was the Dutch version of the Dizziness Handicap Inventory (DHI) (32, 33) at 6 months, revealing whether the patient had developed chronic dizziness or not. Twenty-five questions, that can be answered with yes (four points), sometimes (two points) or no (zero points), assess possible functional, emotional, and physical impairments due to dizziness. The higher the score (maximum 100), the worse the perceived handicap is present: 0–30 equals a low handicap, 31–60 a moderate handicap, and over 60 a severe handicap (23).

#### Koos classification

All VS were diagnosed before surgery by Magnetic Resonance Imaging and tumor size was graded based on the Koos classification (34, 35): grade 1 (small intracanalicular tumor), grade 2 (small tumor with protrusion into the cerebellopontine angle, no contact with the brainstem), grade 3 (tumor occupying the cerebellopontine cistern with no brainstem displacement), and grade 4 (large tumor with brainstem and cranial nerve displacement) (32, 33).

#### Preoperative vestibular function

Before surgery, the vestibular function was assessed by binaural bithermal caloric testing and the sinusoidal harmonic acceleration test. The complete testing procedure was described elsewhere (31, 36). Based on the slow phase velocity (°/s) of the caloric nystagmus, obtained during the maximal response of a caloric irrigation, Jongkees' formula was used to calculate the percentage of labyrinth asymmetries. Labyrinth asymmetry was considered normal if the difference between both ears was < 19% (36). Based on the slow phase velocity component during sinusoidal harmonic acceleration, vestibulo-ocular reflex (VOR) gain and VOR phase were calculated. The VOR gain was considered low if < 0.29 and a delay in the system was indicated by a VOR phase above 18°. Based on our normative data, 95% confidence intervals for VOR gain and phase were, respectively, (0.29; 0.87) and (−1.4; 18.1). (36). Labyrinth asymmetry, VOR gain, and VOR phase were used in the statistical analysis.

#### Treatment protocol

In the period between 2001 and 2007, patients after VS surgery were engaged in three different treatment protocols (31, 37): (1) the General Instructions-group received education concerning the vestibular system and general instructions to be physically as active as possible, (2) the Vestibular Rehabilitation-group received the same content as group 1 with an additional customized vestibular rehabilitation home-exercise program, and (3) the Vestibular Rehabilitation + Baclofen-group received the same content as group 2 with an additional medical therapy program with Baclofen up to 6 weeks after surgery (31, 37). During the follow-up sessions, the patients of treatment groups 2 and 3 were able to ask advice concerning their vestibular rehabilitation program. During the retrospective data collection period, two prospective clinical studies were conducted at the time (31, 37). In the first study, patients were randomly assigned to group 1 or 2 by a closed envelope (31). In the second study, patients were asked to participate in group 3. In case patients refused to participate in group 3, they were assigned to group 2 as this was standard care at the time (37). Patients that were seen in between or after the two studies performed the second treatment protocol (group 2). The vestibular rehabilitation program was the same for both clinical trials. The patients of groups 2 and 3 reported a daily exercise duration of ~ 30 min. Treatment adherence was high up to 9 weeks after surgery but decreased between the 9th and 12th week. We observed that 2 months after surgery, most patients had returned to their daily activities, which probably resulted in less time available and less need to perform the exercise program.

#### Physical activity level

All patients were advised to be physically as active as possible after surgery. To estimate the patient's actual activity level, 9 weeks after surgery, the patient was asked to subjectively score his or her level of physical activity during the past week. Physical activity was scored using a visual analogue scale, ranging from zero physical activity (0) to vigorous physical activity (100). The following examples were given to the patients to help them score their level of physical activity: 0 means you spend all day in your bed while 100 indicates the highest level of physical activity you can imagine.

#### Balance performance

Static and dynamic balance performance was assessed 9 weeks post-surgery. Static balance assessment consisted of 4 different static balance test conditions with eyes closed: Romberg with Jendrassik maneuver, standing on foam, tandem stance, and single leg stance. The patient was asked to stand in each position for a maximum of 30 s. Timing was stopped earlier, in case of loss of balance or change in foot position. Each condition was repeated three times, unless the maximum score of 30 s was already reached during the first or second trial. A standing balance sum was calculated by adding the best scores, leading to a maximum possible score of 120 s (38). Dynamic balance was assessed using the Timed Up and Go test. Patients were asked to sit on a chair. After the signal “start,” patients had to stand up, walk 3 m, turn 180° to walk back to the chair and return to sitting position. The patient was instructed to perform this task in a fast but safe manner. The time (seconds) to complete the task was measured from the “start” signal to the moment the patient sat down again with their back touching the back of the chair. Patients performed the Timed Up and Go test 3 times and the best score (shortest time) was withheld (39).

### Statistical analysis

The demographics and study variables were described using means and standard deviations for continuous variables and frequencies for the categorical variables. Afterwards, patients were divided into a group with chronic dizziness (DHI score > 30) and a group without chronic dizziness (DHI score ≤ 30) (23, 40, 41). All study variables were compared between both groups by performing independent samples *t*-tests for the continuous variables (age, preoperative vestibular function (labyrinth asymmetry, VOR gain, and VOR phase), static and dynamic balance performance, and subjective level of physical activity) and Fisher's exact tests for the categorical variables (sex, treatment group, and Koos Classification). Level of significance was set at a *p*-value of 0.05 and effect sizes (Hedges g) were calculated (42–44). An effect size was interpreted as strong when over 0.5 and the 95% confidence interval did not contain zero (45).

To unravel predictive factors for developing chronic dizziness, a multiple linear regression analysis was performed with the DHI score at 6 months as dependent variable. When taking into account the rule of thumb in regression, a maximum of one independent variable per 10 participants was set, besides intercept and slope (46). For the regression analysis, a complete dataset was available for 44 patients. Data were missing since not all patients were able to attend every session or not all outcome variables were gathered for each patient. Therefore, a maximum of 3 independent variables were used in the regression analysis. To identify the 3 main predictive variables for this model, a univariate regression analysis with DHI at 6 months was performed beforehand for all study variables. Ultimately, the 3 variables with the highest *R*^2^ and a *p*-value lower than 0.05 were used in the backward multiple regression analysis. Independent variables were removed from the model if the probability of F (*p*-value) was ≥ 0.10. Multicollinearity was controlled for by calculating the variance inflation factor (VIF). A VIF below five was interpreted as no risk for multicollinearity (47). IBM Statistics SPSS 27 for Windows was used to perform all data analyses.

## Results

### Study participants

A total of 66 patients were included and assessed both preoperatively and after 9 weeks and 6 months. Participants were 50.16 ± 10.91 years old of which 28 were women and 38 were men. All preoperative tumors were graded based on the Koos classification: grade 1 (9 patients), grade 2 (33 patients grade), grade 3 (16 patients), and grade 4 (8 patients). As not all outcome variables were collected for each patient, data were missing concerning preoperative vestibular function (19 patients concerning labyrinth asymmetry and 16 patients concerning VOR gain and VOR phase), treatment group (post-surgery, 2 patients), the level of physical activity (9 weeks, 21 patients), and balance performance (9 weeks, 4 patients).

### Comparison between chronic dizzy and non-chronic dizzy patients

The chronic dizziness and non-chronic dizziness groups consisted of 17 and 49 patients, respectively, meaning that 25.8% of the patients developed chronic dizziness. The differences between the two groups concerning the study variables are shown in Tables 1, 2. Three variables, namely level of physical activity (*p* < 0.001), timed up and go test (*p* = 0.041), and standing balance performance (*p* = 0.023), showed significantly better scores in the non-chronic dizziness group. Effect sizes for the continuous variables were presented as well, with strong effect sizes for the level of physical activity (Hedges g = 1.270 [0.577; 1.952]), timed up and go test (Hedges g = 0.588 [0.025; 1.147]), and standing balance performance (Hedges g = 0.587 [0.024; 1.146]).

**Table 1:** Comparison of continuous variables between non-chronic dizziness and chronic dizziness groups.

	**Non-chronic dizziness group**	**Chronic dizziness group**	**Independent samples *t*-test**	**Effect sizes**
	**Mean (SD)**	**Mean (SD)**	***P*-value**	**Hedges g**
	***N* = 49**	***N* = 17**		**(95% CI)**
Age (years)	49.19 (11.30) *N* = 49	52.95 (9.48) *N* = 17	0.224	0.341 (−0.208; 0.889)
Timed Up and Go test (s)	7.78 (1.16716) *N* = 45	8.53 (1.43833) *N* = 17	0.041^*^	0.588 (0.025; 1.147)^*^
Standing balance performance (s)	57.41 (20.25) *N* = 45	46.07 (15.29) *N* = 17	0.023^*^	0.587 (0.024; 1.146)^*^
Subjective level of physical activity (mm, max 100)	77.16 (16.56) *N* = 32	53.54 (22.06) *N* = 13	<0.001^*^	1.270 (0.577; 1.952)^*^
Preoperative vestibular function (labyrinth asymmetry)	45.77 (27.30) *N* = 35	39.17 (20.88) *N* = 12	0.449	0.251 (−0.896; 0.397)
Preoperative vestibular function (VOR gain)	0.45 (0.23) *N* = 36	0.38 (0.20) *N* = 14	0.327	0.307 (−0.305; 0.916)
Preoperative vestibular function (VOR phase)	19.11 (13.41) *N* = 36	18.00 (10.54) *N* = 14	0.782	0.086 (−0.522; 0.694)

*N, number; SD, standard deviation; s, seconds; mm, millimeter; max, maximum*.

* *Significant result*.

**Table 2:** Comparison of categorical variables between chronic dizziness and non-chronic dizziness groups.

		**Non-chronic dizziness group**	**Chronic dizziness group**	**Fischer's exact test**
		***N* = 49**	***N* = 17**	***P*-value**
Sex	Male	23	5	0.262
	Female	26	12	
Koos classification	Grade 1	6	3	0.737
	Grade 2	25	8	
	Grade 3	11	5	
	Grade 4	7	1	
Treatment group	General instructions	12	6	0.592
	Customized VR	26	8	
	Customized VR and baclofen	10	2	

*VR, vestibular rehabilitation*.

### Predictive factors for perceived disability due to dizziness at 6 months

The univariate regression analyses revealed that the level of physical activity (*R*^2^ = 0.166; b = −0.404; *p* = 0.005), timed up and go test (*R*^2^ = 0.110; b = 4.929; *p* = 0.008), and standing balance performance (*R*^2^ = 0.109; b = −0.321; *p* = 0.009) explained the largest variance in dizziness complaints compared to the other studied variables (Table 3). Thereafter, a multiple backward regression analysis was performed with these 3 variables. After the elimination process, 2 variables (level of physical activity and timed up and go test) remained in the model which significantly predicted the DHI-score at 6 months and explained up to 23.9% of the variance in DHI-score (*F*_2,42_ = 6.581; *R*^2^ = 0.239; *p* = 0.003). However, the two variables are not independent prognostic factors for chronic dizziness. A collinearity analysis was performed for the regression model, indicating no risk of multicollinearity (VIF = 1.289 for the level of physical activity, VIF = 1.498 for timed up and go test, and VIF = 1.341 for static balance performance).

**Table 3:** Predictive factors for perceived disability due to dizziness at 6 months.

**Univariable regression analyses with perceived disability at 6 months as the dependent variable**				
**Independent variable**	***R* ^2^**	**Intercept (a)**	**Slope (b)**	**Level of significance**
Age	0.032	3.022	0.302	*p* = 0.153
Sex	0.031	14.357	6.590	*p* = 0.155
Koos classification	0.009	23.017	−2.072	*p* = 0.438
Preoperative vestibular function (LA)	0.022	21.764	−0.101	*p* = 0.324
Preoperative vestibular function (VOR gain)	0.010	21.374	−8.017	*p* = 0.484
Preoperative vestibular function (VOR phase)	0.001	18.951	−0.053	*p* = 0.799
Treatment group	0.008	20.130	−2.384	*p* = 0.494
Standing Balance Performance^*^	0.109	35.984	−0.321	*p* = 0.009^*^
Timed Up and Go test^*^	0.110	−20.795	4.929	*p* = 0.008^*^
Subjective level of physical activity^*^	0.166	47.961	−0.404	*p* = 0.005^*^
**Multiple regression analysis with perceived disability at 6 months as the dependent variable**				
**Model**	***R* ^2^**	***F*_x,y_**	**Level of significance**	
Model after elimination with two variables^*^	0.239	*F*_2,42_ = 6.581	*p* = 0.003^*^	
**Independent variable**	**Intercept (a)**	**Slope (b)**	**Level of significance**	
Timed Up and Go test	−1.836	5.173	*p* = 0.052	
Subjective level of physical activity		−0.268	*p* = 0.081	

Perceived disability = DHI-score at 6 months, *R*^2^ = explained variance of the dependent variable, intercept (a) and slope/regression-coefficient (b) in regression formula: Y (DHI-score) = a + bX(independent variable).

LA, labyrinthine asymmetry; VOR, Vestibulo-Ocular Reflex; *F*, ratio of the mean regression sum of squares divided by the mean error sum of squares; x/y, degrees of freedom.

* Significant result (*p* < 0.05).

## Discussion

### Summary of the results

The objective of this study was to explore the association between the perceived level of physical activity during the subacute phase among other variables and chronic dizziness after a VS resection. Patients with chronic dizziness 6 months after surgery showed lower levels of physical activity and poorer balance performance 9 weeks after surgery compared to patients who did not become chronically dizzy. Furthermore, the perceived level of physical activity and dynamic balance performance explained up to 23.9% of the variance in the DHI score at 6 months. Other factors such as age, sex, Koos classification, treatment group, and preoperative vestibular function did not show significant associations with chronic dizziness. In summary, these results identify the perceived level of physical activity and balance performance during the subacute phase as possible prognostic factors for developing chronic dizziness after a VS resection.

The majority of the study variables — age, sex, Koos classification, preoperative vestibular function, and treatment group — were not significantly associated with chronic dizziness. In the literature, the relation of these factors with dizziness was studied in patients with dizziness, unsteadiness, or balance problems (48–50). Similar to the previously mentioned studies in patients after VS resection (7, 13, 14), conflicting results were found concerning the relation of these factors with dizziness: one study reported a significant association with sex (48) and two other studies reported no significant associations with sex (49), age (48, 49), or vestibular function (50). Besides the significant association between dizziness and sex (48), these results are thus similar to what was found in this study. Furthermore, our results confirmed that physical activity and thus exposure to movement is required to stimulate central vestibular compensation after an acute unilateral vestibular deafferentation (15–19). However, the literature revealed that, in the long-term, levels of physical activity in these patients remain lower, compared to physical activity levels in healthy adults (27–29). Our results also unraveled an association between physical activity and the development of chronic dizziness after VS resection. Although in the literature limited information is available concerning this association, physical activity levels in chronic unilateral vestibulopathy-patients correlated moderately (*r* > 0.4 or *r* < −0.40) (51) with vertigo severity (*r* = −0.602) (27), dizziness severity (*r* = -0.493) (27), dizziness frequency (*r* = -0.487) (27), and challenging static balance performance (*r* = -0.452) (28). Combined with the level of physical activity, balance performance during the subacute phase explained a significant amount of variance in dizziness complaints after 6 months. The possible prognostic value of balance performance after VS resection was, however, not investigated before. Instead of balance performance, the predictive value of psychological factors and visual dependency on chronic dizziness were already explored. For example, VS patients revealed to have elevated levels of preoperative psychological burden, related to the number of present symptoms (52), and VS patients with the presence of psychological factors, such as anxiety or depression, show worse balance performance (53, 54). At the time, we did not systematically assess these factors. However, the presence of psychological factors—namely fear avoidance beliefs—might partially explain the association that was found in this study between physical activity and chronic dizziness. In case of fear of movements that provoke symptoms, insufficient exposure to movement will arise, and thus central compensation is not stimulated as needed, to prevent development into chronic dizziness. The relation between the presence of fear-avoidance beliefs and the level of physical activity was recently studied in a group of patients with mixed vestibular disorders, revealing that the presence of fear-avoidance beliefs significantly predicted activity limitations (55). Another interesting possibly predictive factor to explore is the preoperative amount of visual dependency. VS patients relying more on visual cues for balance control, initially show worse balance performance after surgery (56). Therefore, both presence of psychological factors and type of sensory weighting, for example, greater dependence on visual stimuli (12), seem to influence balance performance. This was confirmed by Cousins et al. who identified both anxiety and visual dependency as prognostic factors for clinical recovery (DHI score after 10 weeks) in vestibular neuritis patients (57). However, the impact of these patient-related factors on chronic dizziness in patients after VS surgery has not been investigated so far. Another preoperative treatment approach for VS, which was not applied in our study, is the preoperative deterioration of vestibular function by, for example, intratympanic gentamicin injection. Preoperative deterioration might stimulate preoperative central vestibular compensation and would perhaps allow clinicians to control the above-mentioned patient-related factors better. However, so far only conflicting results regarding the effect on postoperative symptoms were found (58–62). In summary, large prospective cohort studies in patients after VS resection, investigating the association between multiple objectively measured factors — such as level of physical activity and balance performance on top of other factors such as visual dependence and fear avoidance beliefs — and chronic dizziness, is recommended.

### Clinical implications

Assessing the level of physical activity and balance performance can be rather easily performed, as described in this study. These measurements were conducted in a standardized and safe manner without invasive or time-consuming procedures. However, in contrast to how physical activity was assessed in this study, objective measures should be preferred, such as accelerometers, heart rate monitors, or even smartphone applications (63). Furthermore, measuring the level of physical activity during the subacute phase could raise awareness concerning the actual physical activity level and lead to additional guidance for patients whose activity levels are low. Vestibular rehabilitation might play an important role in the general activation of these patients (64–66). Besides the type of intervention, the timing of both assessment and intervention seems crucial as well, as the 1st weeks after vestibular deafferentation were identified as the most critical time period to stimulate central vestibular compensation (19). Assessing the level of physical activity after 9 weeks, as performed in this study, could, for example, signal that the intensity of vestibular rehabilitation should be increased rather than decreased. In addition, in case of the presence of psychological factors or visual dependency, components such as cognitive behavioral therapy and visual desensitization therapy may need to be added. In our study, the type of treatment was not significantly associated with the development of chronic dizziness. However, customized vestibular rehabilitation might have indirectly influenced the development of chronic dizziness as both variables that were associated with chronic dizziness were more favorable in the patients that received customized vestibular rehabilitation in the first 9 weeks after VS surgery. Indeed, the TUG scores at 9 weeks were higher in the general instruction group (group 1) compared to the modified vestibular rehabilitation groups (groups 2 and 3). In the elderly patients (≥50 years), the difference in TUG scores was significant between both groups with 9.08 (1.17) seconds for the general instruction group and 8.06 (1.12) seconds for the vestibular rehabilitation groups; independent samples *t*-test: *p* = 0.03, while for the entire population this was 8.36 (1.35) seconds for the general instruction group and 7.77 (1.15) seconds for the vestibular rehabilitation groups; independent samples *t*-test: *p* = 0.09 (31). Furthermore, although not significant, a higher level of physical activity was found in the customized vestibular rehabilitation groups (groups 2 and 3) compared to the general instruction group (group 1) with scores of 72.50 (21.71) and 63.64 (18.14), respectively (independent samples *t*-test: *p* = 0.229). Again this difference in the level of physical activity was more pronounced in patients older than 50 years of age: 71.64 (21.11) for the vestibular rehabilitation groups and 57.83 (22.26) for the general instructions group; independent samples *t*-test: *p* = 0.17. The small sample sizes might explain why the differences in TUG and activity level were not significant. In addition, it was observed that after 9 weeks, patients' adherence to the customized vestibular rehabilitation decreased and advice related to vestibular rehabilitation was usually stopped after 12 weeks. One might hypothesize that patients reaching a higher level of activity at 9 weeks stayed active in the following months, thereby partly decreasing the risk of developing chronic dizziness. This might explain why the type of treatment (in the first 9 to 12 weeks after VS surgery) was not associated with the development of chronic dizziness at 6 months. As mentioned before, more research is needed to confirm the influence of physical activity and balance performance on chronic dizziness. Thereafter, research concerning additional assessment and treatment can be performed.

### Limitations

This study included a rather small sample size (*n* = 66). However, with 25.8% of the patients developing chronic dizziness, a representative sample was chosen as this number is in line with the literature (7). The small sample size and a varying amount of collected data per patient only allowed 3 independent variables in the regression analysis. At the ENT department, the self-observed level of physical activity was only requested from the patients from November 2002. Therefore, 21 out of 66 data points were missing concerning the level of physical activity at 9 weeks. When comparing the group with and without available perceived physical activity levels, the DHI score at 6 months did not differ between both. Therefore, the results of this study were thought not to be influenced by these missing data. Another limitation was that the measurement of physical activity in our study was patient-reported and therefore subjective. Furthermore, no information concerning the preoperative level of physical activity was gathered. It is possible that patients with higher levels of physical activity post-surgery might have been physically more active before surgery than those with lower physical activity levels. This preoperative level of physical activity, therefore, might influence the development of chronic dizziness as well (20) and was not taken into account in our study. Other possibly influencing variables, such as presence and duration of (vestibular) symptoms before surgery or location of tumor origin, were not assessed for their role on chronic dizziness as these data were not collected in this study. The lack of these variables could clarify why only 23.9% of the variance in DHI score was explained by our regression model. Finally, although an association was found between the level of physical activity and chronic dizziness, based on this study, no conclusion can be made concerning a possible causal relationship between both. Further longitudinal research is necessary to clarify if physical activity is a prognostic factor for the development of chronic dizziness after VS resection.

## Conclusion

This study revealed associations between (1) the level of physical activity and balance performance during the subacute phase and (2) chronic dizziness after a VS resection. Despite the fact that only 23.9% of the variance of the DHI score at 6 months was explained by the subjective perception of activity level and functional balance at 9 weeks and that the two variables were not independent factors for chronic dizziness, it seems worthwhile to investigate this further and to determine whether objective measures of physical activity can predict chronic dizziness.

## Data availability statement

The data is the property of the University of Antwerp. Requests to access these datasets should be directed to LVa, lien.vanlaer@uantwerpen.be.

## Ethics statement

The studies involving human participants were reviewed and approved by Ethical Committee of the Antwerp University Hospital and University of Antwerp. The patients/participants provided their written informed consent to participate in this study.

## Author contributions

LVa: conception, content, design, conduct, analysis and interpretation of data, presentation of the research, and writing of the manuscript. AH and VV: conception, content, design, interpretation of data, presentation, and critical revision of the research. CD: acquisition of data and critical revision of the research. PV: concept, acquisition of data, and critical revision of the research. LVe: conception, content, design, acquisition and interpretation of data, and presentation and critical revision of the research. All authors contributed to the article and approved the submitted version.

## Funding

This work was supported by the University of Antwerp Research Council (grant number ID 42186), the University of Antwerp, and the Antwerp University Hospital.

## Conflict of interest

The authors declare that the research was conducted in the absence of any commercial or financial relationships that could be construed as a potential conflict of interest.

## Publisher's note

All claims expressed in this article are solely those of the authors and do not necessarily represent those of their affiliated organizations, or those of the publisher, the editors and the reviewers. Any product that may be evaluated in this article, or claim that may be made by its manufacturer, is not guaranteed or endorsed by the publisher.
