# Erratum: Subjective perception of activity level: a prognostic factor for developing chronic dizziness after vestibular schwannoma resection?

**DOI:** 10.3389/fneur.2023.1253462

**Published:** 2023-07-13

**Authors:** Frontiers Production Office

**Affiliations:** Frontiers Media SA, Lausanne, Switzerland

**Keywords:** vestibular schwannoma, physical activity, chronic dizziness, balance, risk factors

Due to a production error, there was a mistake in Table 3 as published. Following a correction requested during production, a change was carried out to the upper part of the table (Univariable regression analyses) and not the lower part of the table (multivariate regression). The corrected Table 3 appears below.

**Table 3:** Predictive factors for perceived disability due to dizziness at 6 months.

**Univariable regression analyses with perceived disability at 6 months as the dependent variable**				
**Independent variable**	***R* ^2^**	**Intercept (a)**	**Slope (b)**	**Level of significance**
Age	0.032	3.022	0.302	*p* = 0.153
Sex	0.031	14.357	6.590	*p* = 0.155
Koos classification	0.009	23.017	−2.072	*p* = 0.438
Preoperative vestibular function (LA)	0.022	21.764	−0.101	*p* = 0.324
Preoperative vestibular function (VOR gain)	0.010	21.374	−8.017	*p* = 0.484
Preoperative vestibular function (VOR phase)	0.001	18.951	−0.053	*p* = 0.799
Treatment group	0.008	20.130	−2.384	*p* = 0.494
Standing Balance Performance^*^	0.109	35.984	−0.321	*p* = 0.009^*^
Timed Up and Go test^*^	0.110	−20.795	4.929	*p* = 0.008^*^
Subjective level of physical activity^*^	0.166	47.961	−0.404	*p* = 0.005^*^
**Multiple regression analysis with perceived disability at 6 months as the dependent variable**				
**Model**	***R* ^2^**	***F*_x,y_**	**Level of significance**	
Model after elimination with two variables^*^	0.239	*F*_2,42_ = 6.581	*p* = 0.003^*^	
**Independent variable**	**Intercept (a)**	**Slope (b)**	**Level of significance**	
Timed Up and Go test	−1.836	5.173	*p* = 0.052	
Subjective level of physical activity		−0.268	*p* = 0.081	

Perceived disability = DHI-score at 6 months, *R*^2^ = explained variance of the dependent variable, intercept (a) and slope/regression-coefficient (b) in regression formula: Y (DHI-score) = a + bX(independent variable).

LA, labyrinthine asymmetry; VOR, Vestibulo-Ocular Reflex; *F*, ratio of the mean regression sum of squares divided by the mean error sum of squares; x/y, degrees of freedom.

* Significant result (*p* < 0.05).

The publisher apologizes for this mistake. The original article has been updated.

